# Characteristic proteins in the plasma of postoperative colorectal and liver cancer patients with Yin deficiency of liver-kidney syndrome

**DOI:** 10.18632/oncotarget.21735

**Published:** 2017-10-11

**Authors:** Qing Ji, Wenhai Wang, Yunquan Luo, Feifei Cai, Yiyu Lu, Wanli Deng, Qi Li, Shibing Su

**Affiliations:** ^1^ Research Center for Traditional Chinese Medicine Complexity System, Shanghai University of Traditional Chinese Medicine, Shanghai 201203, China; ^2^ Department of Medical Oncology, Shuguang Hospital, Shanghai University of Traditional Chinese Medicine, Shanghai 201203, China; ^3^ Department of Medical Oncology, Shuguang Hospital Affiliated Baoshan Branch, Shanghai University of Traditional Chinese Medicine, Shanghai 201901, China; ^4^ Department of Liver and Gallbladder surgery, Shuguang Hospital, Shanghai University of Traditional Chinese Medicine, Shanghai 201203, China

**Keywords:** systems biology, proteomics, traditional Chinese medicine, colorectal cancer, liver cancer

## Abstract

Systems biology and bioinformatics provide the feasibility for the basic research associated with “same traditional Chinese medicine (TCM) syndrome in different diseases”. In this study, the plasma proteins in postoperative colorectal (PCC) and postoperative liver cancer (PLC) patients with YDLKS (Yin deficiency of liver-kidney syndrome) were screened out using iTRAQ combined with LC-MS/MS technology. The results demonstrated that, KNG1, AMBP, SERPING1, etc, were all differentially expressed in both PCC and PLC patients with YDLKS, and associated closely with complement and coagulation cascades pathway. C7 and C2 were another two representative factors involving in former pathway. Further validation showed that, the C7 levels were increased significantly in PLC (*P* < 0.05) and PCC (*P* < 0.05) with YDLKS group compared to those of NS (no obvious TCM syndromes) group. The AMBP levels were down-regulated significantly in PLC with YDLKS group compared to those of PCC with YDLKS group (*P* < 0.05). The significant differences of SERPING1 levels (and C2 levels) were shown between YDLKS and NS in PCC (*P* < 0.01). There were also significant differences of C2 levels between PCC and PLC patients with YDLKS (*P* < 0.05). Moreover, significant differences of C2 levels were also found between PLC and PCC patients with YDLKS (*P* < 0.01). ROC curves indicated that, C7 and SERPING1 independently had a potential diagnostic value in distinguishing YDLKS from NS in PLC and PCC, providing the evidences for the material basis of “same TCM syndrome in different diseases” in PCC and PLC patients with YDLKS.

## INTRODUCTION

In recent years, due to changes in diet and lifestyle, the incidence of colorectal cancer (CRC) increased year by year [1]. Stage IIB or higher (late-stage) CRC patients, or stage II CRC patients with high risk factors, have about 50% possibility to generate recurrence or metastasis within 2 years after surgery, radical chemotherapy and/or radiotherapy. Currently, the concept of Traditional Chinese medicine (TCM) syndromes, also known as ZHENG in Chinese, is an important component of TCM theory. Generally, the TCM syndrome is a clinical profile of signs and symptoms reflecting the inherent pathological changes of various diseases, which helps identifying human body patterns and guiding TCM treatments with TCM herbs [2]. In TCM, one type of cancer may show different TCM syndromes, while different cancers may have an identical syndrome [3]. With regard to CRC patients, there are Dampness-heat syndrome (DHS), Internal retention of toxin stagnation syndrome (IRTSS), Spleen deficiency syndrome (SDS), Yin deficiency of liver-kidney syndrome (YDLKS), Qi and blood deficiency syndrome (QBDS), and Yang deficiency of spleen and kidney syndrome (YDSKS) [4]. After the surgery and several rounds of chemotherapy, most of the CRC patients often show the YDLKS [5].

On the other hand, epidemiological survey of malignant tumor demonstrates that, due to the hepatitis B virus infection and other reasons, the incidence of liver cancer (LC) hovers in the third place, and the mortality rate takes the second place. Recurrence and metastasis are the main problems after first therapy [1]. Previous studies have shown that, the most common TCM syndromes in LC are Qi stagnation and blood stasis syndrome (QSBSS), Liver stagnation and spleen deficiency syndrome (LSSDS), YDLKS, Liver stagnation and Qi stagnation syndrome (LSQSS), SDS, and DHS [4, 6, 7]. Because most of the LC patients have chronic liver disease history, longer duration, as well as postoperative chemotherapy infusion and embolotherapy, the YDLKS is often appeared in those patients according to our clinical observation.

In terms of the postoperative CRC or LC patients, the disease factors are not completely eliminated after surgery, and the postoperative recurrence or metastasis is the main problem to be overcome. TCM syndromes are the core foundation of disease knowing, clinical diagnosis and efficacy evaluation in TCM clinical practice [2, 3, 4]. It shows that, under the theoretical guidance of the treatment based on TCM syndrome differentiation, TCM has obvious characteristics and advantages in the therapy of the complex and refractory diseases like malignant tumors [8]. Of which, the “same syndrome in different diseases” and “same treatment in different diseases” reflect the characteristics of treatment based on TCM syndrome differentiation [9]. However, to further improve the application potential of TCM in the prevention and treatment of malignant tumors, the bottleneck that “interpretation of the biological substance of diseases and TCM syndromes” needs to be broken. Recently, the development of systems biology [10] and bioinformatics [11] provides the feasibility for the basic research associated with “same TCM syndrome in different diseases”.

In recent years, the iTRAQ (isobaric tags for relative and absolute quantitation) technology provides an effective method for quantitative and qualitative detection of low abundance proteins [4]. In this study, we aim to screen out the characteristic proteins in the plasma of postoperative colorectal (PCC) and postoperative liver cancer (PLC) patients with YDLKS using iTRAQ combined with liquid chromatography-tandem mass spectrometry (LC-MS/MS) technology, and to explore the biological foundation of “same syndrome in different diseases”, providing a scientific basis for the “same treatment in different diseases”.

## RESULTS

### Analysis of plasma proteins in PCC and NS patients with YDLKS

**Figure 1:**
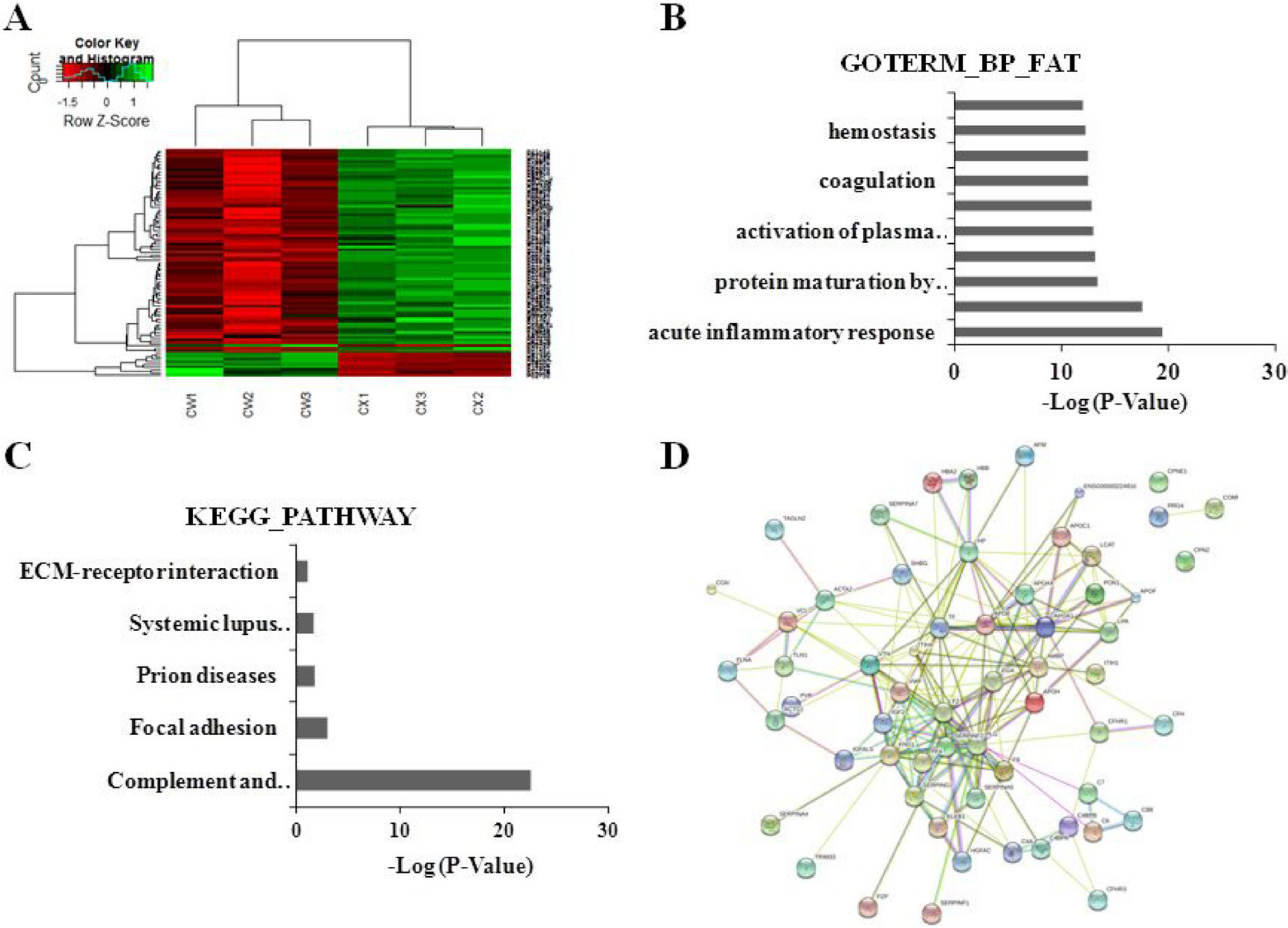
Clustering analyses of DEPs, functional and pathway annotation, and protein-protein interaction analysis in PCC patients with YDLKS compared to those of NS (**A**) Heatmap analysis for the DEPs detected by iTRAQ combined with LC-MS/MS technologies. CW1, CW2 represented the sample from two PCC patients with YDLKS, respectively. CW3 represented the pooled samples from eight PCC patients with YDLKS. CX1, CX2 represented the samples from two PCC patients with NS, respectively. CX3 represented the pooled sample from eight PCC patients with NS. (**B**) GO classification (biological process) of the DEPs using DAVID functional annotation tool. (**C**) KEGG pathway annotation of the DEPs using DAVID functional annotation tool. (**D**) PPI network construction for the DEPs using the STRING database.

Using iTRAQ combined LC-MS/MS technologies, the plasma proteins in 10 PCC patients with YDLKS and 10 PCC patients with NS were detected. The DEPs were identified when the ratio was more than 1.5 with a *t*-test *P*-value cutoff < 0.05. The heatmap was constructed using Partek^®^ Genomics Suite^®^ software. Compared to NS, 85 DEPs were indentified in the PCC patients with YDLKS (Supplementary Materials), including Plasminogen (PLG), Prothrombin F2, Apolipoprotein E (APOE), Kininogen 1 (KNG1), Von Willebrand factor (vWF), Apolipoprotein A1 (APOA1), Vitronectin (VTN), Alpha-2-antiplasmin (SERPINF2), and Insulin-like growth factor II (IGF2), etc (Figure 1A).

The functional classifications of DEPs mainly involved in the acute inflammatory response, response to wounding, complement activation, and so on (Figure 1B). The pathway annotation of DEPs mainly involved in the complement and coagulation cascades, systemic lupus erythematosus, focal adhesion, prion diseases, and so on (Figure 1C).

For more comprehensive understanding of the functional organization of the DEPs, we constructed a protein-protein interaction (PPI) network using the STRING database. 60 DEPs were involved in PPI, in which 52 proteins were up-regulated, and the other 8 proteins were down-regulated. In the up-regulated proteins, several proteins like PLG, F2, APOE, KNG1, HP, TF, SERPINF2 and IGF2 have strong connections with other proteins (Figure 1D, Supplementary Figure 1). These proteins have important functions in biological regulation, coagulation system, etc.

### Analysis of plasma proteins in PLC and NS patients with YDLKS

**Figure 2:**
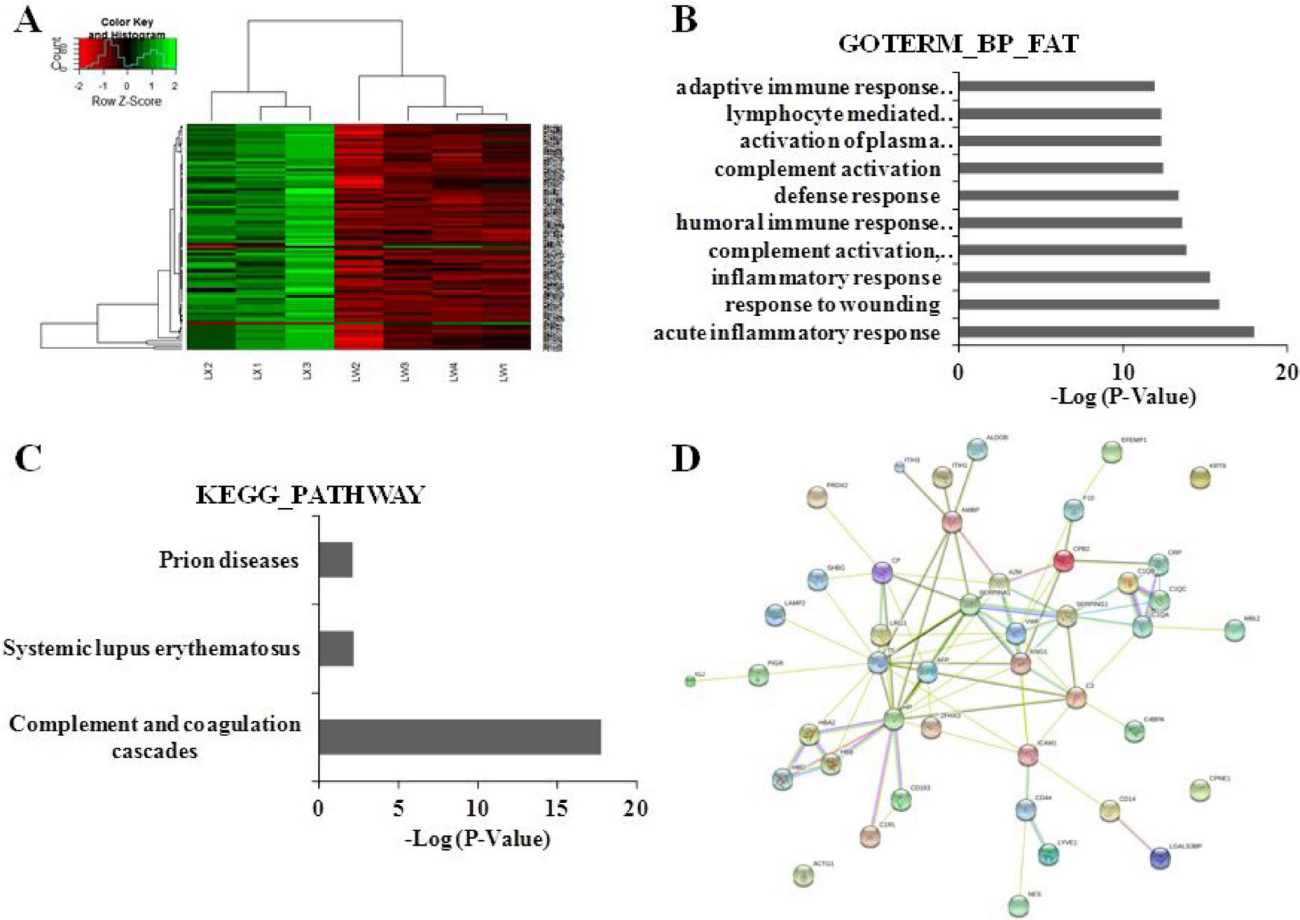
Clustering analyses of DEPs, functional and pathway annotation, and protein-protein interaction analysis in PLC patients with YDLKS compared to those of NS (**A**) Heatmap analysis for the DEPs detected by iTRAQ combined with LC-MS/MS technologies. LW1, LW2, LW3 represented the sample from three PLC patients with YDLKS, respectively. LW4 represented the pooled sample from seven PLC patients with NS. LX1, LX2 represented the sample from three PLC patients with NS, respectively. LX3 represented the pooled sample from eight PLC patients with NS. (**B**) GO classification (biological process) of the DEPs using DAVID functional annotation tool. (**C**) KEGG pathway annotation of the DEPs using DAVID functional annotation tool. (**D**) PPI network construction for the DEPs using the STRING database.

Using iTRAQ combined LC-MS/MS technologies, the plasma proteins in 10 PLC patients with YDLKS and 10 PLC patients with NS were also detected. The DEPs were screened out according to the criterion in PCC. Compared to NS, 91 DEPs were identified in the PLC patients with YDLKS (Supplementary Materials), including vWF, KNG1, Alpha-1-antitrypsin (SERPINA1), Complement C3 (C3), Alpha-2-macroglobulin (A2M), Plasma protease SERPING1, Intercellular adhesion molecule 1 (ICAM1), Alpha-1-microglobulin/bikunin precursor (AMBP), and Ceruloplasmin (CP), etc (Figure 2A).

The functional classifications of DEPs mainly involved in the acute inflammatory response, complement activation, humoral immune response, inflammatory response, and so on (Figure 2B). The pathway annotation of DEPs mainly involved the complement and coagulation cascades, systemic lupus erythematosus, focal adhesion, prion diseases, and so on (Figure 2C). The PPI network analysis showed that, 44 DEPs were involved protein-protein interaction, in which 41 proteins were up-regulated, and the other 3 proteins were down-regulated. In the up-regulated proteins, several proteins like A2M, C1QA, KNG1, HP, TF, SERPINA1, SERPING1, AMBP, C3, CP, ICAM1 and VWF have strong connections with other proteins (Figure 2D, Supplementary Figure 2). These proteins also have important functions in biological regulation, coagulation system, etc.

### Common DEPs in PCC and PLC patients with YDLKS

**Figure 3:**
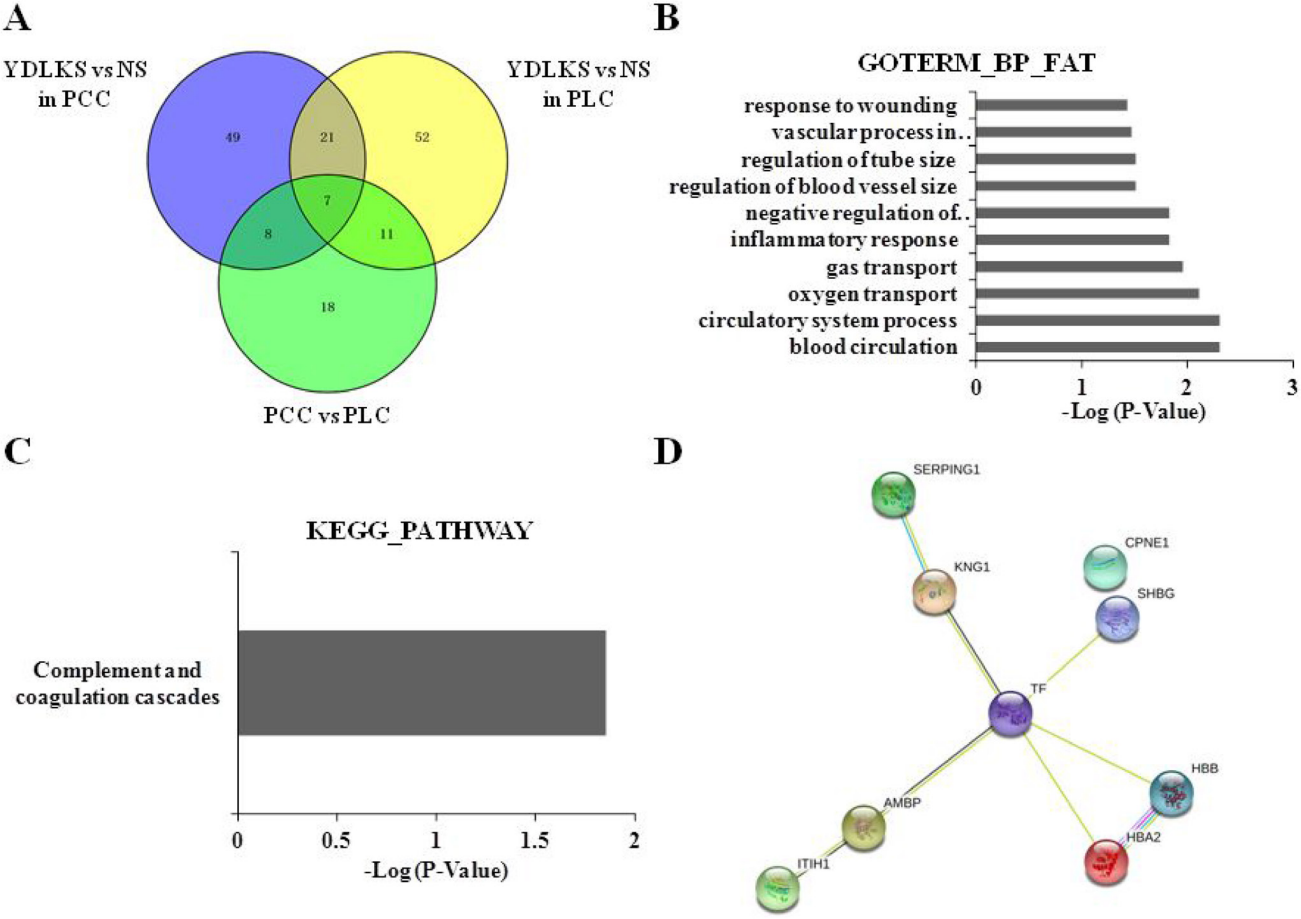
Clustering analyses of common DEPs in PCC and PLC, protein-protein interaction analysis, and functional and pathway annotation (**A**) Venn diagrams analysis of the Common DEPs in PCC and PLC patients with YDLKS in compared to those of NS. (**B**) GO classification (biological process) of the DEPs using DAVID functional annotation tool. (**C**) KEGG pathway annotation of the DEPs using DAVID functional annotation tool. (**D**) PPI network construction for the DEPs using the STRING database.

By Venn diagrams analysis, 21 common DEPs (Figure 3A, Supplementary Table 1) were screened out from the plasma of PCC and PLC patients with YDLKS, including 9 distinct up-regulated proteins like KNG1, Hemoglomin subunit alpha2 (HBA2), Hemoglomin subunit beta (HBB), AMBP, Sex hormone-binding globulin (SHBG), Carboxypeptidase N catalytic chain (CPN1), and SERPING1.

### Functional classifications of identified DEPs

**Figure 4:**
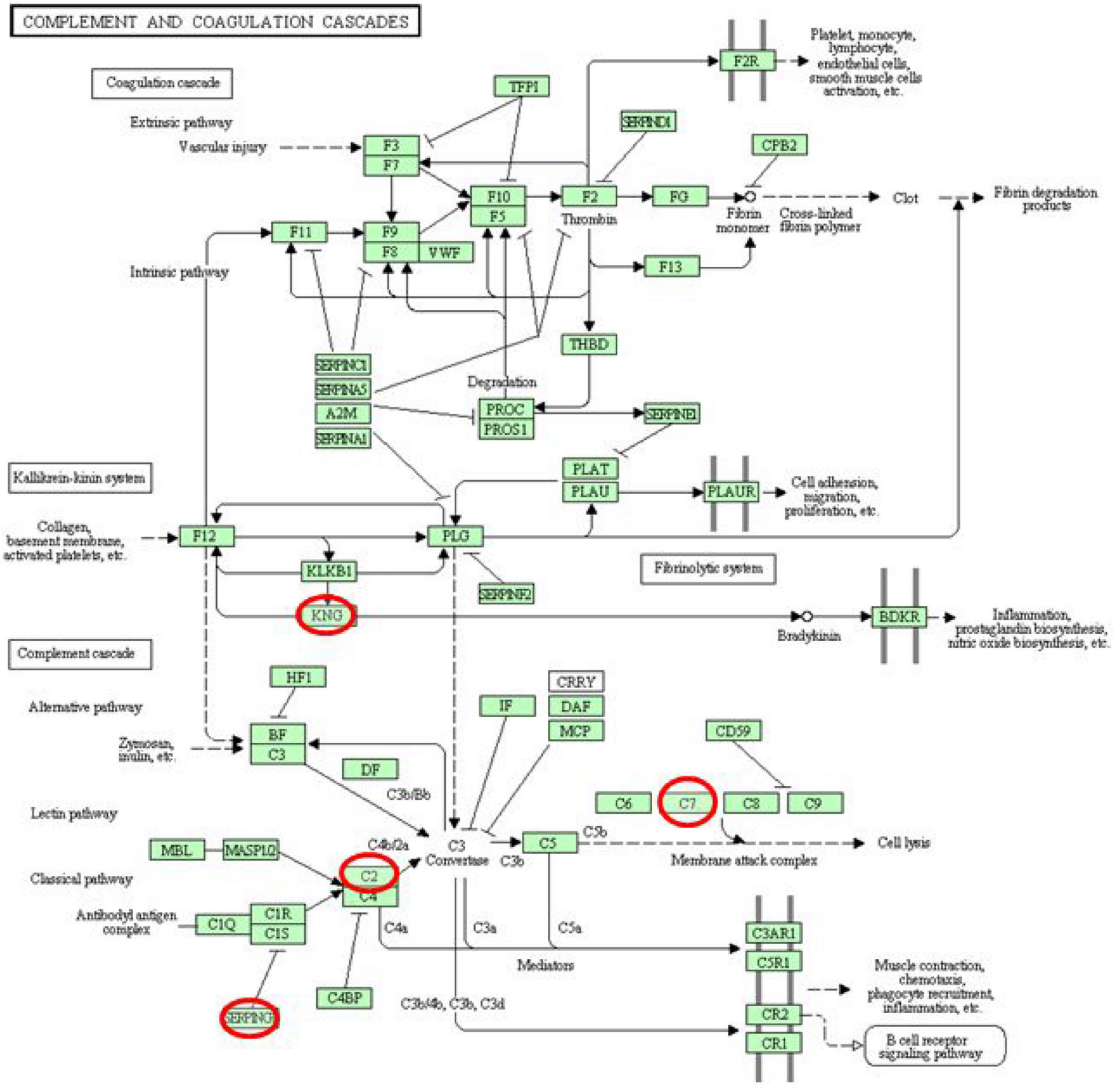
General drawing of the complement and coagulation cascade pathway

DAVID functional annotation tool was used to determine the functional classifications of identified proteins. Enrichment analysis using biological processes indicated that the 9 common up-regulated proteins mainly involved in the blood circulation, circulatory system process, oxygen transport, gas transport, inflammatory response, negative regulation of immune response, regulation of blood vessel size, and so on (Figure 3B). For KEGG analysis, we found that, several DEPs including KNG1, AMBP, and SERPING1 have important associations with complement and coagulation cascade pathway (Figure 3C). The PPI network analysis for 9 common up-regulated proteins showed that, taking TF as the center, other 8 proteins established close links with it (Figure 3D, Supplementary Figure 3). From the 21 common DEPs in Supplementary Table 1, we also found several proteins such as C2 and C7 were closely connected with complement and coagulation cascades pathway (Figure 4).

### ELISA validation

**Figure 5:**
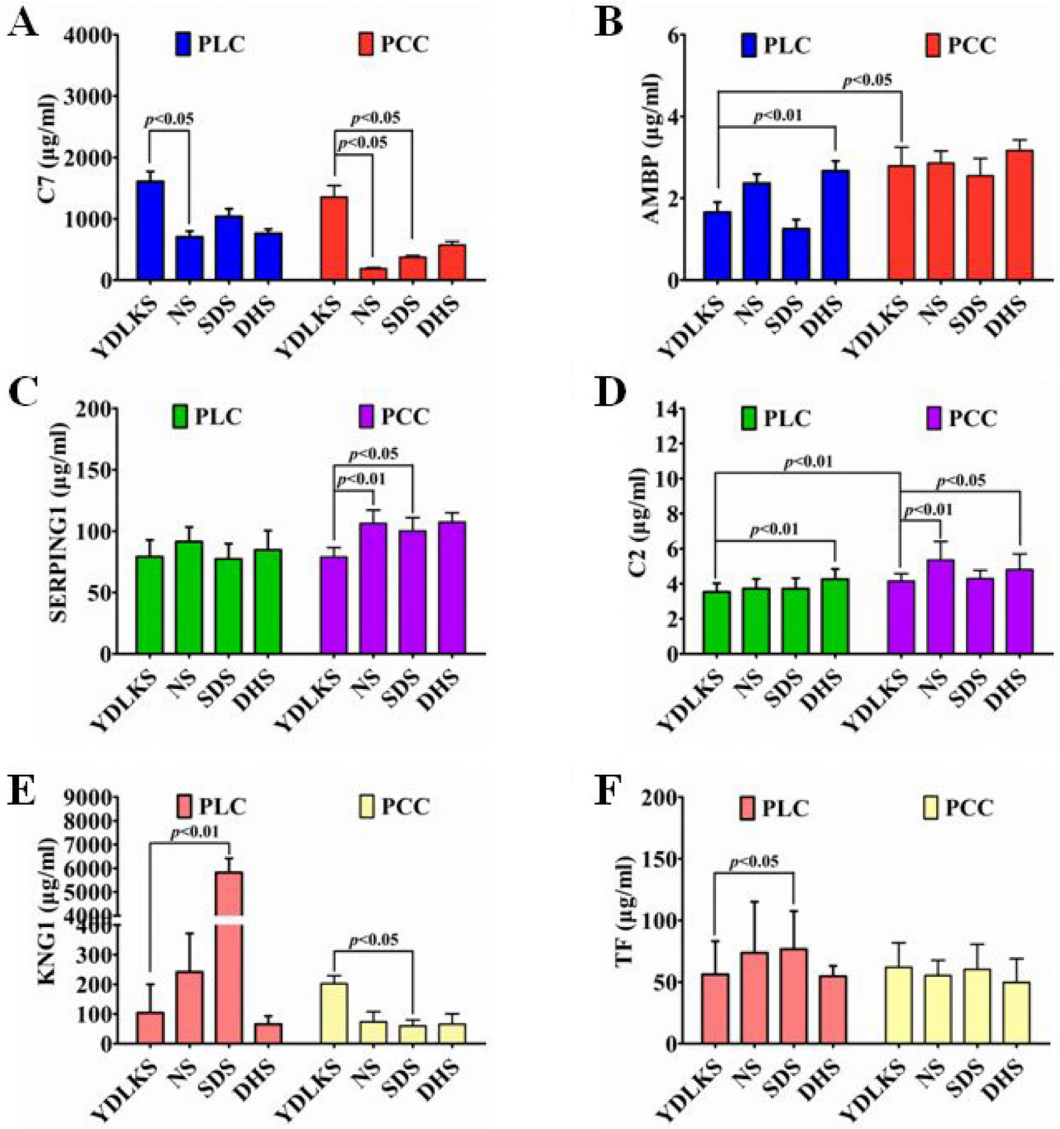
Plasma C7, AMBP, SERPING1, C2, KNG1 and TF levels in PCC and PLC patients with YDLKS and NS were analyzed by ELISA measurement (**A**) Plasma C7 levels. (**B**) Plasma AMBP levels. (**C**) Plasma SERPING1 levels. (**D**) Plasma C2 levels. (**E**) Plasma KNG1 levels. (**F**) Plasma TF levels. Each group, *n* = 30. *P* < 0.05 was considered as statistically significant, and *P* < 0.01 was considered as statistically highly significant.

Basing on the results of Venn diagrams analysis, functional enrichment, KEGG pathway analysis and PPI network analysis for the common DEPs in PCC and PLC patients with YDLKS, the levels of SERPING1, KNG1, AMBP, TF, C2 and C7 in human plasma were detected by ELISA. All these DEPs have important association with complement and coagulation cascade pathway, and show close connection among them. For ELISA measurements, 30 samples from each group were used, and all the standard curves resulted in a curve fit with an R2 of 0.99. As shown in Figure 5A, the plasma levels of C7 was increased significantly in PLC (*P* < 0.05) and PCC (*P* < 0.05) with YDLKS group compared to those of NS group. Whether in PLC or PCC, C7 levels were much higher in YDLKS group than those in SDS and DHS group, especially that there were significant differences between the YDLKS group and SDS group in PCC (*P* < 0.05). However, there was no significant difference in the levels of C7 between PLC patients with YDLKS and PCC patients with YDLKS (*P* > 0.05). We also found that, there were no significant differences of the AMBP levels (Figure 5B) in PLC and PCC with YDLKS compared to those of NS group (*P* > 0.05), but the AMBP levels were much higher in PLC with YDLKS than those in PLC with DHS (*P* < 0.01). Nevertheless, the AMBP levels were down-regulated significantly in PLC with YDLKS group compared to those in PCC with YDLKS group (*P* < 0.05). In addition, we found that there was significant difference in the levels of AMBP between PLC patients with SDS and PCC patients with SDS (*P* < 0.01, data not shown).

The expression levels of SERPING1 (Figure 5C) and C2 (Figure 5D) were both down-regulated in PCC and PLC with YDLKS group compared to those of NS group (*P* > 0.05). Moreover, significant differences of SERPING1 and C2 levels were found between PCC with YDLKS and PCC with NS (*P* < 0.01). With regard to C2, there was significant difference between PCC patients with YDLKS and PLC patients with YDLKS (*P* < 0.05), except SERPING1 (*P* > 0.05). Additionally, we also found that, the SERPING1 levels were lower in PCC with YDLKS than those in PCC with SDS (*P* < 0.05), and the C2 levels were lower in PLC and PCC with YDLKS than those in PLC and PCC with DHS (*P* < 0.01, *P* < 0.05, respectively). Moreover, significant difference of C2 levels was also found between PLC and PCC patients with YDLKS (*P* < 0.01).

For KNG1 and TF (Figure 5E, 5F), no significant differences were found between the PLC or PCC patients with YDLKS and those of NS group (*P* > 0.05). But overall, the plasma levels of KNG1 or TF were both down-regulated in PLC with YDLKS group compared to those of NS group (*P* > 0.05), while the plasma levels of KNG1 or TF were both up-regulated in PCC with YDLKS group compared to those of NS group (*P* > 0.05). Moreover, the plasma levels of KNG1 were down-regulated significantly in PLC with YDLKS compared to those of PLC with SDS (*P* < 0.01), while the plasma levels of KNG1 were up-regulated in PCC with YDLKS compared to those of PCC with SDS (*P* < 0.05). In addition, the plasma levels of TF were also down-regulated in PLC with YDLKS compared to those of PLC with SDS (*P* < 0.05). However, there was no significant difference in the levels of KNG1 or TF between PLC and PCC patients with YDLKS (*P* > 0.05).

Preliminary validation results indicated that, plasma C7 and SERPING1 may be valuable characteristic proteins for both PLC and PCC patients with YDLKS. C2 may be one of the valuable characteristic proteins for PCC patients with YDLKS. In PCC, plasma C7, SERPING1 and KNG1 may be the characteristic proteins to make a distinction between YDLKS and SDS, and plasma C2 may be the characteristic protein to make a distinction between YDLKS and DHS. In PLC, plasma KNG1 and TF may be the characteristic proteins to make a distinction between YDLKS and SDS, and plasma AMBP and C2 may be the characteristic proteins to make a distinction between YDLKS and DHS. In addition, the plasma levels of AMBP and C2 may be characteristic proteins to make a distinction between PLC with YDLKS and PCC with YDLKS.

### ROC curve analysis

**Figure 6:**
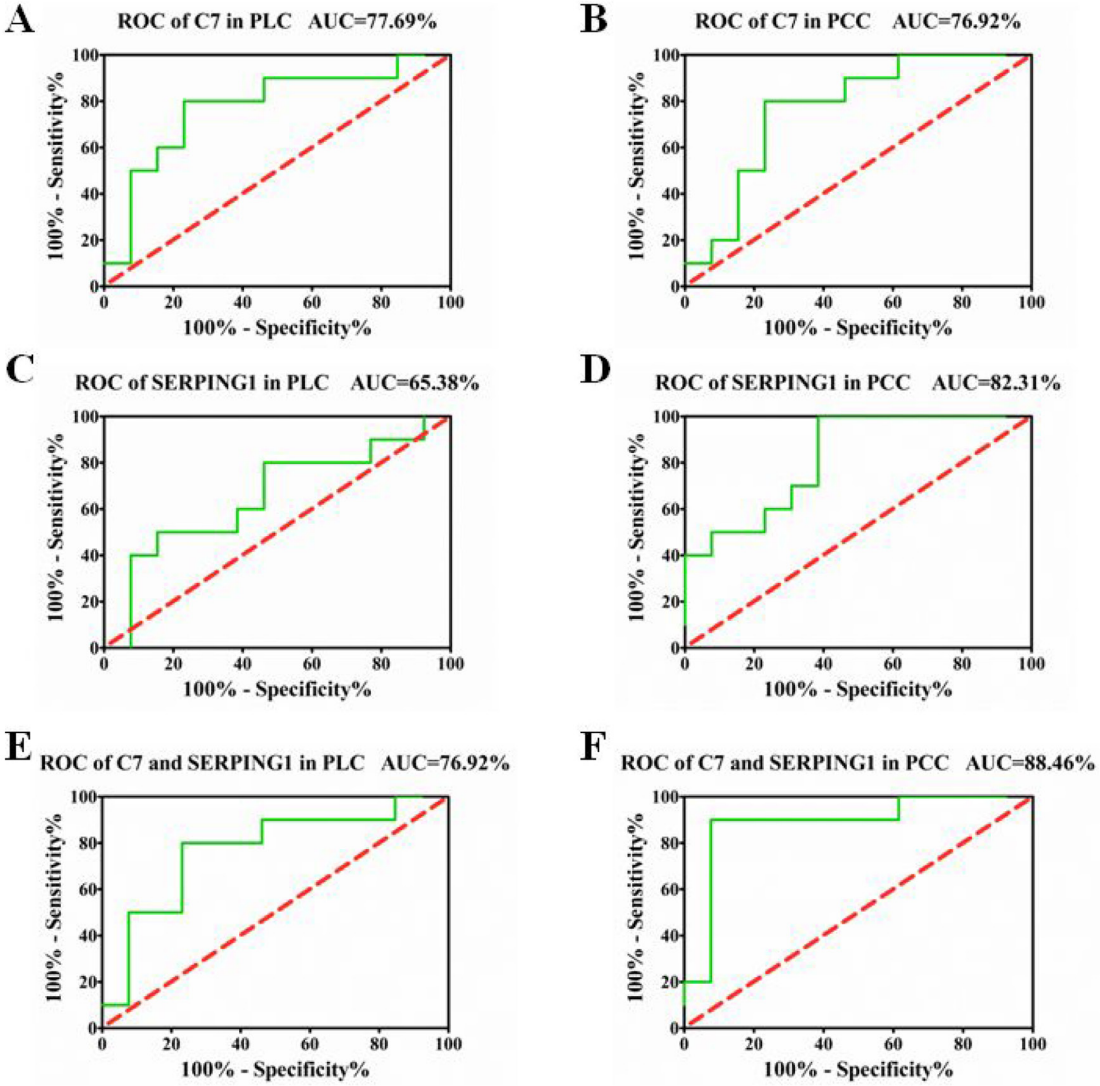
ROC curves for PLC and PCC patients with YDLKS and NS based on C7 and SERPING1 plasma levels (**A**) ROC curve for PLC patients with YDLKS and NS based on C7 plasma levels. (**B**) ROC curve for PCC patients with YDLKS and NS based on C7 plasma levels. (**C**) ROC curve for PLC patients with YDLKS and NS based on SERPING1 plasma levels. (**D**) ROC curve for PCC patients with YDLKS and NS based on SERPING1 plasma levels. (**E**) Combined ROC curve for PLC patients with YDLKS and NS based on C7 and SERPING1 plasma levels. (**F**) Combined ROC curve for PCC patients with YDLKS and NS based on C7 and SERPING1 plasma levels. The areas under ROC curve (AUC) showed high accuracy above 0.9, good accuracy between 0.7–0.9, lower accuracy between 0.5 and 0.7, no diagnostic value at 0.5 or below.

To explore whether C7 and SERPING1 can serve as a potential biomarker to distinguish YDLKS from NS in PLC and PCC, we constructed ROC curves by grouping plasma YDLKS samples into one class and all NS samples into another class. For C7 in PLC and PCC, the areas under ROC curve (AUC) were 0.7769 and 0.7692 (Figure 6A, 6B), respectively. For SERPING1 in PLC and PCC, the AUC were 0.6538 and 0.8231 (Figure 6C, 6D), respectively. However, for combined detection of C7 and SERPING1 in PLC and PCC, the AUC were 0.7692 and 0.8846 (Figure 6E, 6F), respectively. Above ROC curves implied that, C7 and SERPING1 independently had a potential diagnostic value in distinguishing YDLKS from NS in PLC and PCC, and combined detection of C7 and SERPING1 showed more high accuracy in distinguishing YDLKS from NS in PCC.

## DISCUSSION

In recent years, the incidence of primary CRC (PCC) increased year by year, and more of which were diagnosed as advanced PCC, only 10% were diagnosed as Stage I PCC patients. Although there was follow-up chemotherapy or radiotherapy after surgery, the rates of recurrence and metastasis were still high [1]. Because the majority of the PCC patients received several cycles of chemotherapy, YDLKS were more often seen in those patients, and usually accompanying with the SDS [5].

Primary LC (PLC) is due to the carcinogenesis of the liver cells or the intrahepatic bile duct epithelial cells [12]. Compared with other countries around the world, the mortality of PLC in China ranks the first, and the 5-year survival rate of PLC in the world is 19%, but only 13% in China [1]. TCM believes that, PLC develops by the following process: depressed emotions, heat toxin, Qi stagnation, improper diet and other internal injuries give rise to the disharmony of liver and spleen, and further aggravate the phlegm and blood stasis, which gradually accumulate and form a blockage in the flank. Qi and blood deficiency is the cause of PLC, and mutual stasis of heat and toxin is the pathomechanism of LC [3, 4, 6, 7]. Generally, PLC patients have a history of chronic liver disease and longer duration, often receive postoperative chemotherapy infusion and embolization, which lead to the occurrence of YDLKS in those patients accompanying with SDS that shows poor appetite, abdominal distension and diarrhea [4].

For PCC or PLC patients, postoperative recurrence and metastasis are the main problems need to be overcome [13, 14]. Based on the characteristics of treatment based on TCM syndrome differentiation that “same TCM syndrome in different diseases” and “same treatment in different diseases” [9], to further improve the application potential of cancer prevention and treatment using Chinese medicine, we have to break the bottleneck that “interpretation of the biological substance of diseases and TCM syndromes”. In recent years, the systems biology technologies such as transcriptomics [15], proteomics [16] and metabolomics [17] provide very good technical conditions for TCM research in view of “same syndrome in different diseases” and “same treatment in different diseases”.

A variety of gene expression profiling, metabolic profiling and differentially expressed materials were found in the TCM syndrome classification research of rheumatoid arthritis [18], hepatitis B or cirrhosis [9, 19, 20], and other diseases [21]. Using microarray technology and gas chromatography-mass spectrometry (GC/MS) combined with pattern recognition methods, our group found that the differentially gene expression profiles [9], protein fingerprinting spectrum [19] and urine-metabolite characteristic spectrum [20] among different TCM syndromes in hepatitis B patients or cirrhosis, and established the effect evaluation methods between diseases and TCM syndromes based on differences in gene signaling pathway and metabolite.

iTRAQ proteomics technology is an isotope labeling technique launched by Applied Biosystems in USA at year 2004, which solves the problems that the qualitative and quantitative detection of low-abundance proteins [4]. Compared to the traditional qualitative and quantitative proteomics methods, iTRAQ technology can perform absolute and relative quantitative simultaneously using four or eight kinds of samples with good reproducible. Additionally, isotope labeling method can accurately grasp the dynamic changes of proteins. Currently, iTRAQ technology is also used to find targets of TCM in the treatment of different diseases. Sundaramurthi et al. [22] confirmed that, Tianma can regulate the metabolism of differentiated neuroblastoma cell by changing the expression of 38 proteins. Moreover, the effect of Tianma on correcting the function and activity of Tpi1, Ppia, Ncam1, Uchl1, Septin-2 and Hsp90aa1 proteins, provided conclusive evidence for Tianma in the treatment of seizures, hypertension and headaches [22]. However, the proteomics investigation associated with “same TCM syndrome in different diseases” in PCC and PLC has not been seriously reported by far.

In this study, using iTRAQ combined with LC-MS/MS technology, more than 9 significantly up-regulated proteins were identified from the PCC and PLC patients with YDLKS in compared to those of NS, including KNG1, HBA2, HBB, AMBP, SHBG, CPN1, TF, ITIH1, SERPING1, etc. Clustering analyses showed that above 9 distinct DEPs mainly involved in the blood circulation, circulatory system process, oxygen transport, gas transport, inflammatory response, etc. And KEGG analysis demonstrated that, several DEPs such as KNG1, AMBP, SERPING1, C2 and C7 have important association with complement and coagulation cascade pathway. To validate the iTRAQ results of KNG1, AMBP, SERPING1, C2 and C7, ELISA measurement was performed. Both in PCC and PLC, a significant increase of the C7 and a decrease of SERPING1 were observed in the YDLKS group compared to those of the NS group. The ELISA validation of C7 in 30 samples was in agreement with the iTRAQ results. However, the ELISA results of SERPING1 in 30 samples were not coherent with the iTRAQ results, indicating that the general expression of SERPING1 in plasma was decreased in PCC and PLC with YDLKS comparing with NS, which needs more samples to validate in the future. Complement components are mainly produced in the liver and play very key roles in innate immunity [23]. In the assembly of the terminal complement components, C7 plays a pivotal role because its attachment to C5b-6 leads to the initial insertion of the complex into cell membrane. Furthermore, cytolytically inactive C5b-7 has chemotactic activity for polymorphonuclear leucocytes and induces further cellular inflammatory response as well as C5b-9 [24]. However, recent reports suggest that, C7 are highly expressed in HCC tissues, and C7 combined with CFH protein control the stemness of liver cancer cells via LSF-1 [25].

SERPING1, a protease inhibitor approved by the FDA for the treatment of hereditary angioedema, serves as regulators for complement activation. SERPING1 is able to inactivate several fibrinolytic and coagulation system proteases, and can also suppresses inflammation via inhibiting both the complement and contact (kallikrein-kinin) systems [26]. Although the mechanisms and functional effects of complement-specific deregulation on the tumor microenvironment are still unclear, the concept of blocking complement for the treatment of cancer is gaining recognition. Therefore, complement-related anticancer strategies are a promising challenge and provide good prospects for successful treatment of cancers [27]. An analysis of the proteomic signature of ovarian cancer tumor fluid also showed that SERPING1 was statistically significant between benign and malignant group [28].

Moreover, in PCC, plasma C7, SERPING1, KNG1 and C2 may be the characteristic proteins to make a distinction between YDLKS and SDS or between YDLKS and DHS. In PLC, plasma KNG1, TF, AMBP and C2 may be the characteristic proteins to make a distinction between YDLKS and SDS or between YDLKS and DHS. In addition, the plasma levels of AMBP and C2 may be characteristic proteins to make a distinction between PLC with YDLKS and PCC with YDLKS. Except C7 and SERPING1, KNG1, AMBP and C2 also played important roles in complement and coagulation cascade pathway. KNG1 belongs to kininogen protein family [29]. It is a multifunctional protein, and plays different roles in a variety of pathological and physiological processes, such as fibrinolysis, thrombosis, inflammation, and tumor formation [30]. AMBP belongs to the superfamily of lipocalin transport proteins and may play a role in the regulation of inflammatory processes [31]. Complement component C2 is a serum glycoprotein that functions as part of the classical pathway of the complement system. In details, activated C1 cleaves C2 into C2a and C2b, and the serine proteinase C2a then combines with complement factor 4b to create the C3 or C5 convertase [32].

To understand the function and potential mechanism of the DEPs, we constructed a protein-protein interaction (PPI) network. We found that the 9 common up-regulated proteins mainly involved the blood circulation, circulatory system process, oxygen transport, gas transport, inflammatory response, negative regulation of immune response, regulation of blood vessel size. Several DEPs such as KNG1, AMBP and SERPING1 have important association with complement and coagulation cascade pathway. The PPI network analysis also showed that, taking TF as the center, other 8 proteins, especially the complement and coagulation cascades pathway associated proteins like KNG1, AMBP and SERPING1 established close links with TF.

In conclusion, this study demonstrated that, iTRAQ combined with LC-MS/MS technologies could be effectively applied in differential proteomic analysis. We found that plasma C7 and SERPING1 may be potential diagnostic biomarkers for PCC and PLC patients with YDLKS, providing the evidence of the material basis of “same TCM syndrome in different diseases” in PCC and PLC patients with YDLKS. C7 and SERPING1 independently have a potential diagnostic value in distinguishing YDLKS from NS in PLC and PCC, while combined detection of C7 and SERPING1 enhances the accuracy in distinguishing YDLKS from NS in PCC. In the future, increasing patients’ number and evaluating the dynamic changes of C7 and SERPING1 following treatment based on YDLKS identification in PCC and PLC patient, will greatly enhance the application potential of C7 and SERPING1 in the clinical diagnosis. Although the mechanism of these identified proteins remains to be examined, our results have brought about a better understanding of the molecular mechanism of “same TCM syndrome in different diseases” through the proteomics approach.

## MATERIALS AND METHODS

### Clinical materials

Clinical data were collected from PLC or PCC patients in Shuguang Hospital, Shanghai University of TCM from 2014 to 2015 years. PLC patients were those without metastases by physical and biochemical examination after surgery or radiofrequency and other local treatment, as well as the assured cytology examination and the postoperative pathology confirmation. PCC patients are those without metastases by physical and biochemical examination, as well as the diagnosis by the postoperative pathology confirmation. All the collected cancer patients were fit with the YDLKS, 18 to 85 years old, male or female, KPS (Karnofsky) ≥ 60 points, will survive more than 3 months, and signed informed consent. We had access to information that could identify individual participants during or after data collection. All the human materials were obtained with informed consent, and this project was approved by the Clinical Research Ethics Committee of Shuguang Hospital, Shanghai University of Traditional Chinese Medicine.

### Inclusion criteria

For PLC and PCC patients with YDLKS, the diagnostic criteria refer to “Chinese medicine clinical research guidelines” (third edition). YDLKS shows the symptoms that blurred vision, xerophthalmia, tinnitus, skin itching, dry mouth, pale or red tongue, poor or no fur, poor libido, constipation, soreness and flaccidity of waist and knees, rapid pulse and vexing heat in the chest, palms and soles. The controls in this study were chosen from the PLC or PCC patients with no obvious TCM syndromes (NS), which were no complaints and no obvious signs. SDS and DHS were also chosen as the control. SDS shows the symptoms that vomiting, diarrhea, edema, bleeding, amenorrhea, vaginal, cold limbs, infantile polysalivation, etc. DHS shows the symptoms that heavy cumbersome head and body, fever more often in the afternoon, dull fever, no relieve because of sweating, yellow and greasy tongue coating, rapid pulse, etc. All the patients with typical YDLKS were diagnosed by 3 TCM chief physicians.

### Exclusion criteria

(1) does not meet the inclusion criteria, (2) with serious heart, kidney, hematopoietic system disorders and other factors affect the drugs evaluation, (3) with mental disorders, (4) with gastrointestinal obstruction, (5) with poor compliance.

### Collection of the plasma samples

Peripheral blood samples were collected from 30 PCC patients with typical YDLKS, 30 PLC patients with typical YDLKS, 30 PCC patients with NS, and 30 PLC patients with NS, respectively. 1 ml of peripheral blood samples were centrifuged at 14,000g and 4°C for 40 min. The supernatant was collected, and 200 μl aliquot were stored at −80°C.

### Depletion of the high-abundance proteins

All the plasma protein detection and analyses were started from 2016. 40 of 120 samples from PCC patients with typical YDLKS (10), PLC patients with typical YDLKS (10), PCC patients with NS (10) and PLC patients with NS (10) were chosen for characteristic proteins detection using iTRAQ combined with LC-MS/MS technology. Plasma samples were thawed on ice. Equal amounts of plasma from individuals in each group were pooled to yield 2 distinct pools of 500 ml each. To deplete the high-abundance proteins of each plasma pool, a ProteoExtract™ Albumin/IgG Removal Kit (Merck, Germany, Cat 122642) was used according to the manufacture’s instruction. Briefly, plasma was loaded onto the column and proteins bound with high specificity to a bead-based library of diverse peptide ligands. High-abundance proteins which saturated their corresponding ligands were washed out of the column. The remaining low- and mid-abundance proteins in the column were then eluted and collected. After elution, protein was precipitated with acetone overnight at 20°C and dissolved with iTRAQ dissolution buffer. The total protein content of each group was quantified with a Bradford protein assay kit. 10 μg purified samples was taken for SDS-PAGE electrophoresis.

### Protein digestion and peptide iTRAQ labeling

For protein digestion and peptide iTRAQ labeling, the samples were divided into 4 groups as follows: 10 PCC patients with YDLKS, 10 PLC patients with YDLKS, 10 PCC patients with NS, and 10 PLC patients with NS. For PCC, each group with 10 samples was arranged as follows: two separated samples and one pooled samples with 8 different samples together. For PLC with YDLKS, 10 samples were arranged as follows: three separated samples and one pooled samples with 7 different samples together. For PLC with NS, 10 samples were arranged as follows: two separated samples and one pooled samples with 8 different samples together. In view of each sample, 100 mg of the processed protein were reduced, blocked on cysteines, and digested with Trypsin with the ratio of protein: trypsin = 20:1. After trypsin digestion at 37°C for 12 h, the peptides were dried by vacuum centrifugation, following by reconstituting in 0.5 M TEAB and processing according to the manufacturer’s protocol for 8-plex iTRAQ (Applied Biosystems). Briefly, one unit of iTRAQ reagent was thawed and reconstituted in isopropanol. Peptides were then labeled individually with iTRAQ tags. Following incubation for 2 hours at room temperature with the iTRAQ reagent, the labeled samples were equally mixed prior to further analysis. Strong cation exchange chromatography SCX chromatography was performed with a Shimadzu LC-20AB HPLC Pump system. The iTRAQ-labeled peptide mixtures were reconstituted with 4 mL buffer A (25 mM NaH_2_PO_4_ in 25% ACN, pH 2.7) and loaded onto a Ultremex SCX column (4.6 × 250 mm, 5 mm, Phenomenex). The peptides were eluted with a gradient of buffer A for 10 min, 5–35% buffer B (25 mM NaH_2_PO_4_, 1 M KCl in 25% ACN, pH 2.7) for 11 min, 35–80% buffer B for 1 min at a flow rate of 1 mL/min. The system was then maintained at 80% buffer B for 3 min before equilibrating with buffer A for 10 min prior to the next injection. Elution was monitored by measuring the absorbance at 214 nm, and fractions were collected every 1 min. The eluted peptides were pooled into 20 fractions, desalted with a Strata X C18 column and vacuum-dried.

### LC-ESI–MS/MS analysis

Each fraction was resuspended in buffer A (2% ACN, 0.1% FA) and centrifuged at 20,000g for 10 min. In each fraction, the final concentration of peptides was approximately 0.25 mg/mL. Using an auto sampler, 9 mL of supernatant was loaded onto a Symmetry C18 column (180 mm × 20 mm, 5 mm) on a nanoACQuity UPLC system (waters) and eluted with buffer A at 2 mL/min for 15 min. Peptides were then eluted onto a BEH130C18 column (100mm × 10 mm, 1.7 mm) for online trapping, desalting, and analytical separations. At a flow rate of 300 nL/min, the samples were loaded with 5% buffer B (98% ACN, 0.1% FA) for 1 min and eluted with a 40 min gradient from 5 to 35% B, followed by a 5 min linear gradient to 80% B, maintenance at 80% B for 5 min. Initial chromatographic conditions were restored in 2 min.

Data acquisition was performed with a TripleTOF 5600 System (AB SCIEX, Concord, ON) fitted with a Nanospray III source (AB SCIEX, Concord, ON) and a pulled quartz tip as the emitter (New Objectives, Woburn, MA). Data was acquired using an ion spray voltage of 2.5 kV, curtain gas of 30 PSI, nebulizer gas of 5 PSI, and an interface heater temperature of 150°C. For information dependent acquisition, survey scans were acquired in 250 ms and the top 30 product ion scans were collected if exceeding a threshold of 120 counts per second and with a 2+–5+ charge-state. Four time bins were summed for each scan at a pulser frequency value of 11 kHz, through monitoring of the 40 GHz multichannel TDC detector with 4-anode/channel detection. A sweeping collision energy setting of 355 eV was applied to all precursor ions for collision-induced dissociation. Dynamic exclusion was set for 1/2 of peak width (18 s), and then the precursor is refreshed off the exclusion list. The iTRAQ experiments were performed as three technical replicates to gather reliable quantitative information.

### Data analysis

The resulting MS/MS spectra were searched against the IPI human sequence databases with MASCOT software (Matrix Science, London, U.K.; version 2.4). For protein identification and quantification, a peptide mass tolerance of 2 ppm was allowed for intact peptide masses and 0.05 Da for fragmented ions. One missed cleavage was allowed in the trypsin digests. Carbamidomethylation of cysteine was considered a fixed modification, and the conversion of N-terminal glutamine to pyro-glutamic acid and methionine oxidation were considered variable modifications. All identified peptides had an ion score above the Mascot peptide identity threshold, and a protein was considered identified if at least two such unique peptide match was apparent for the protein.

The differentially expressed proteins (DEPs) were identified when the ratio was bigger than 1.5 with a *t*-test *P*-value cutoff < 0.05. The heatmap was constructed using Partek Genomics Suite software, version 6.6 Copyright; 2015, Partek Inc., St. Louis, MO, USA. The functional classifications and pathway annotation of the DEPs were performed the DAVID functional annotation tool (database for annotation, visualization and integrated discovery) (http://david.abcc.ncifcrf.gov). Protein-protein interaction (PPI) network mode was created using the STRING database (http://string-db.org/).

### ELISA analysis

To confirm the identified proteins obtained by mass spectrometry, the protein contents were also measured by ELISA analysis. Commercially available ELISA kits (Abcam Company, USA) and 100 μL samples from PCC and PLC patients with YDLKS, SDS, DHS and NS (each group, *n* = 30) were used for assays. The ELISA analyses were performed in duplicate according to the manufacturer’s instructions.

### Statistical analysis

All the data were presented as mean SD from three independent experiments. Quantitative variables were analyzed by Student’s *t* tests. Receiver operating characteristic (ROC) analysis, binary logistic regression, and Kaplan-Meier analysis were performed with GraphPad Prism 5.0 software. A Venn diagram is a diagram that shows all possible logical relations between a finite collection of different sets. The significance level was set at 5% (*P* < 0.05).

## SUPPLEMENTARY MATERIALS FIGURES AND TABLE


