# Supplementary material for: Characteristic proteins in the plasma of postoperative colorectal and liver cancer patients with Yin deficiency of liver-kidney syndrome
Source: Oncotarget. 2017 Oct 11;8(61):103223–35. doi: 10.18632/oncotarget.21735 (PMC5732723; doi:10.18632/oncotarget.21735)
Supplement: Supplementary file 1 [file oncotarget-08-103223-s001.pdf]

# Characteristic proteins in the plasma of postoperative colorectal and liver cancer patients with Yin deficiency of liver-kidney syndrome

## SUPPLEMENTARY MATERIALS

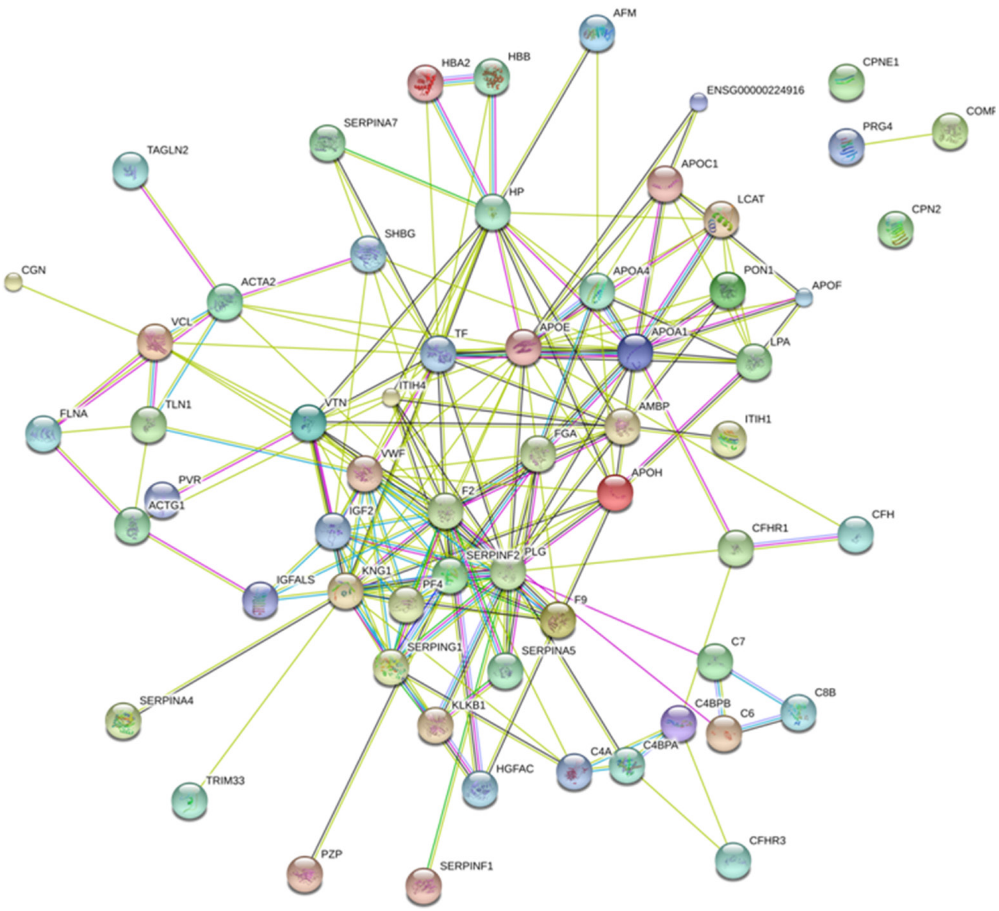


**Supplementary Figure 1: Magnification of Figure 1D.**

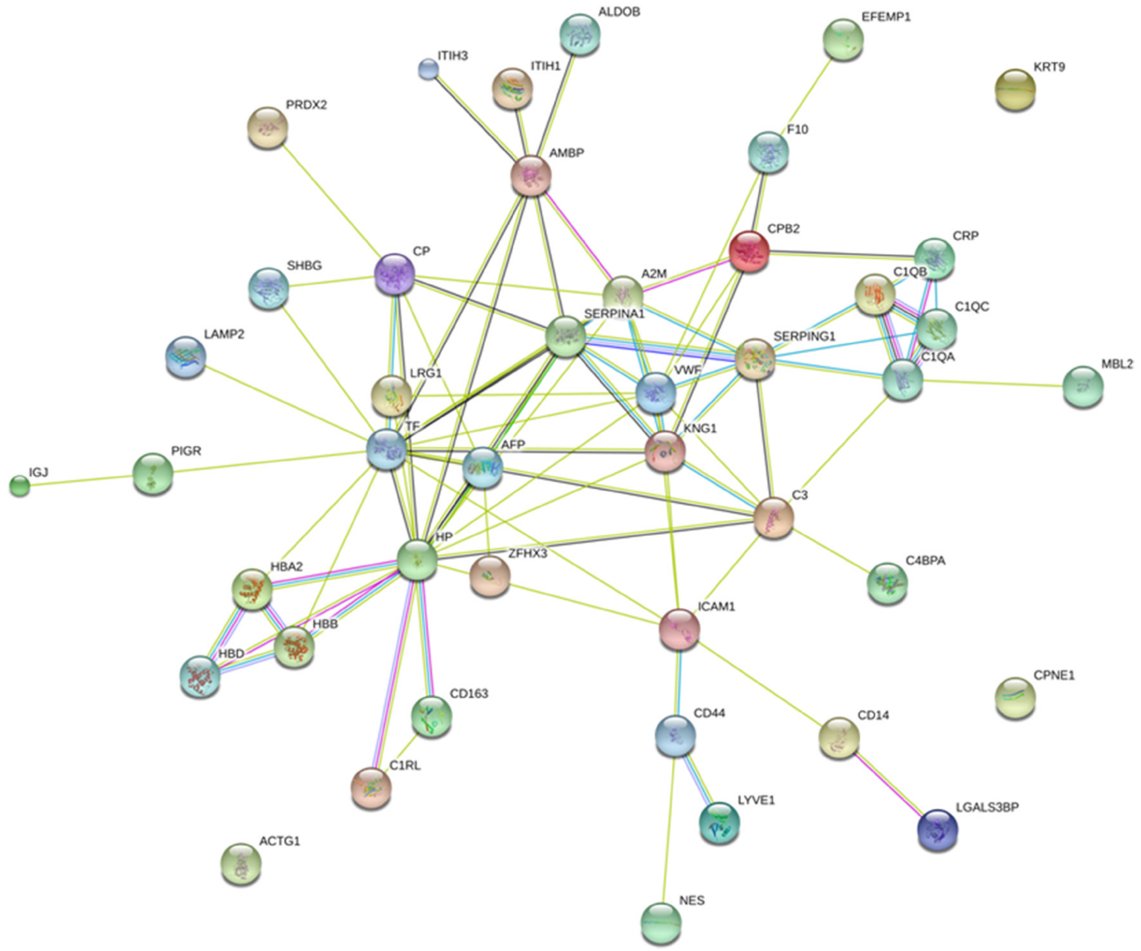


**Supplementary Figure 2: Magnification of Figure 2D.**

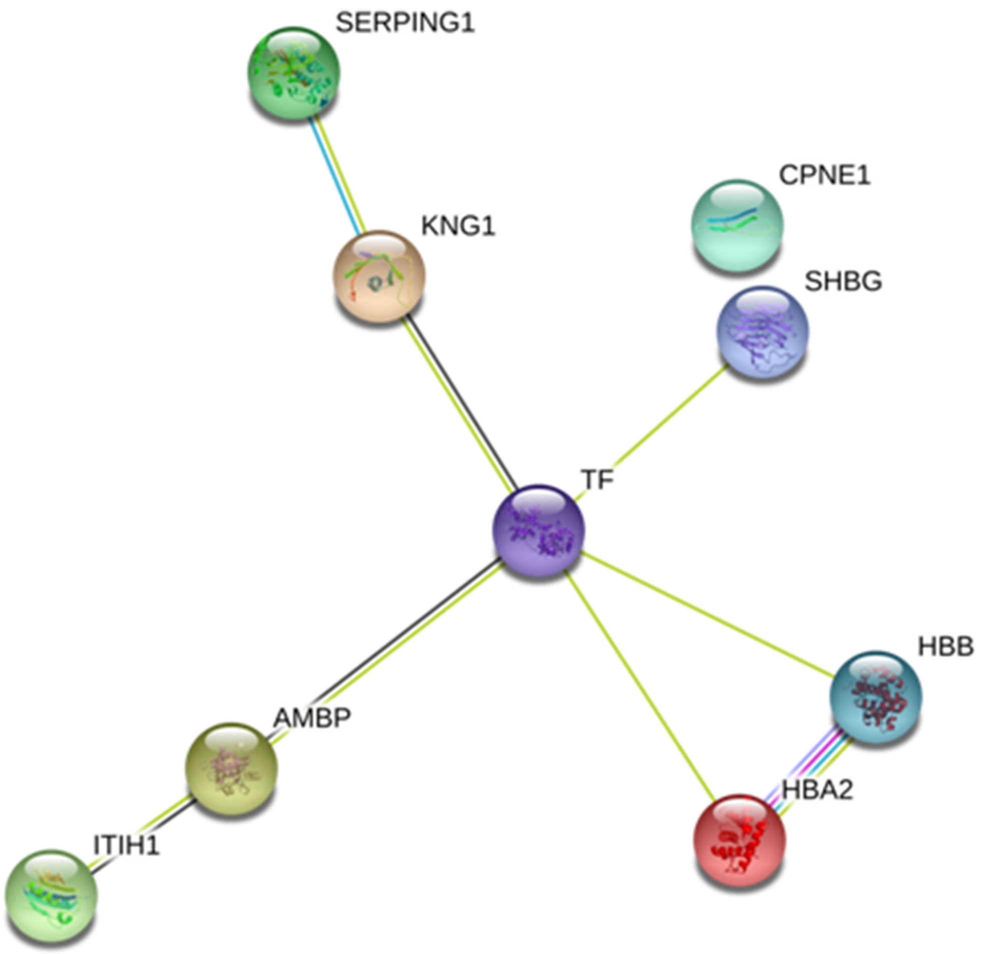


**Supplementary Figure 3: Magnification of Figure 3D.**

**Supplementary Table 1: All the 21 common DEPs in postoperative CRC and LC patients with YDLKS in compared with those of NS**

| Gene ID | Protein names | Gene names |
|---|---|---|
| Q92954 | Proteoglycan 4 (Lubricin) | PRG4 |
| P15169 | Carboxypeptidase N catalytic chain (CPN) | CPN1 |
| Q9P2M7 | Cingulin | CGN |
| P19827 | Inter-alpha-trypsin inhibitor heavy chain H1 | ITIH1 |
| O95445 | Apolipoprotein M (Apo-M) | APOM |
| P04217 | Alpha-1B-glycoprotein | A1BG |
| Q8NET4 | Retrotransposon gag domain-containing protein 1 | RGAG1 |
| O75882 | Attractin (DPPT-L) (Mahogany homolog) | ATRN |
| P68871 | Hemoglobin subunit beta (Beta-globin) | HBB |
| P10643 | Complement component C7 | C7 |
| P02760 | Protein AMBP | AMBP |
| P01042 | Kininogen-1 | KNG1 |
| P69905 | Hemoglobin subunit alpha | HBA1 |
| P06681 | Complement C2 | C2 |
| P02654 | Apolipoprotein C-I (Apo-CI) | APOC1 |
| P05155 | Plasma protease SERPING1 | SERPING1 |
| O75928 | E3 SUMO-protein ligase PIAS2 | PIAS2 |
| P04206 | Ig kappa chain V-III region GOL (Rheumatoid factor) | |
| P02787 | Serotransferrin (Transferrin) | TF |
| Q9Y5Y7 | Lymphatic vessel endothelial hyaluronic acid receptor 1 | LYVE1 |
| P04278 | Sex hormone-binding globulin (SHBG) | SHBG |
